# [*N*′-(4-Dec­yloxy-2-oxido­benzyl­idene)-3-hy­droxy-2-naphtho­hydrazidato-κ^3^ *N*,*O*,*O*′]di­methyl­tin(IV): crystal structure and Hirshfeld surface analysis

**DOI:** 10.1107/S2056989017002365

**Published:** 2017-02-17

**Authors:** Siti Nadiah Binti Mohd Rosely, Rusnah Syahila Duali Hussen, See Mun Lee, Nathan R. Halcovitch, Mukesh M. Jotani, Edward R. T. Tiekink

**Affiliations:** a Department of Chemistry, University of Malaya, 50603 Kuala Lumpur, Malaysia; b Research Centre for Crystalline Materials, School of Science and Technology, Sunway University, 47500 Bandar Sunway, Selangor Darul Ehsan, Malaysia; c Department of Chemistry, Lancaster University, Lancaster LA1 4YB, United Kingdom; d Department of Physics, Bhavan’s Sheth R. A. College of Science, Ahmedabad, Gujarat 380001, India

**Keywords:** crystal structure, organotin, Schiff base, Hirshfeld surface analysis

## Abstract

The title diorganotin compound, [Sn(CH_3_)_2_(C_28_H_32_N_2_O_4_)], features a distorted SnC_2_NO_2_ coordination geometry almost inter­mediate between ideal trigonal–bipyramidal and square-pyramidal. The dianionic Schiff base ligand coordinates in a tridentate fashion *via* two alkoxide O and hydrazinyl N atoms; an intra­molecular hy­droxy-O—H⋯N(hydrazin­yl) hydrogen bond is noted. The alk­oxy chain has an all-*trans* conformation, and to the first approximation, the mol­ecule has local mirror symmetry relating the two Sn-bound methyl groups. Supra­molecular layers sustained by imine-C—H⋯O(hy­droxy), π–π [between dec­yloxy-substituted benzene rings with an inter-centroid separation of 3.7724 (13) Å], C—H⋯π(arene) and C—H⋯π(chelate ring) inter­actions are formed in the crystal; layers stack along the *c* axis with no directional inter­actions between them. The presence of C—H⋯π(chelate ring) inter­actions in the crystal is clearly evident from an analysis of the calculated Hirshfeld surface.

## Abstract 2

A highly distorted penta­coordinated C_2_NO_2_ geometry is observed for the Sn atom owing to a tridentate mode of coordination of the Schiff base ligand and the restricted bite angles it subtends. In the crystal, supra­molecular layers sustained by C—H⋯O, π–π, C—H⋯π(arene) and C—H⋯π(chelate ring) inter­actions are formed.

## Chemical context   

Organotin(IV) compounds with Schiff base ligands have been actively studied because of their versatile chemistry, *e.g*. solution *versus* solid-state structures, and their potential as biologically active compounds such as in anti-cancer and anti-microbial applications (Davies *et al.*, 2008 ▸; Nath & Saini, 2011 ▸). Among these Schiff base ligands, those derived from 3-hy­droxy-2-napthoic hydrazide have long been known to have promising anti-microbial (Dogan *et al.*, 1998*b* ▸) and anti-convulsant activities (Dogan *et al.*, 1998*a* ▸). Subsequently, various organotin compounds derived from these Schiff base ligands have been prepared and their anti-cancer potential explored (Lee *et al.*, 2012 ▸, 2013 ▸). These studies have revealed inter­esting biological activities and often correlations were possible with their solid-state structures (Lee *et al.*, 2009 ▸, 2010 ▸). Complementary studies on vanadium complexes with these Schiff base ligands focused upon their urease inhibitory activities (You *et al.*, 2012 ▸). In addition, the catalytic properties of vanadium (Hosseini-Monfared *et al.*, 2010 ▸, 2014 ▸), cerium (Jiao *et al.*, 2014 ▸) and palladium complexes (Arumugam *et al.*, 2015 ▸) have been explored. Further, structural data for copper (Liu *et al.*, 2012 ▸), molybdenum (Miao, 2012 ▸) and vanadium (Kurup *et al.*, 2010 ▸) complexes are available. As part of our on-going work with these ONO tridentate ligands (Lee *et al.*, 2013 ▸), we hereby describe the crystal and mol­ecular structures of the title compound, (I), as well as a detailed analysis of the inter­molecular associations through a Hirshfeld surface analysis.
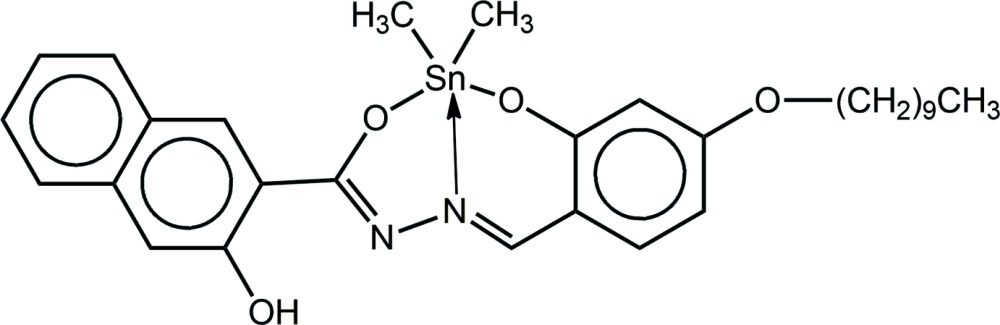


## Structural commentary   

**Figure 1:**
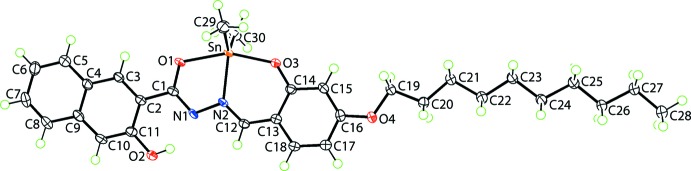
The mol­ecular structure of (I), showing the atom-labelling scheme and displacement ellipsoids at the 70% probability level.

The tin(IV) atom in (I), Fig. 1 ▸, is complexed by a di-anionic, tridentate Schiff base ligand noteworthy for the appended fused-ring system and for the long alk­oxy chain substituent. The five-coordinate geometry is completed by two Sn-bound methyl groups, Table 1 ▸. The resulting C_2_NO_2_ coordination geometry is highly distorted with the value of τ being 0.52, *i.e*. almost exactly inter­mediate between ideal square-pyramidal (τ = 0) and trigonal–bipyramidal (τ = 1.0) (Addison *et al.*, 1984 ▸). The widest angle at the tin atom is subtended by the two alkoxide-O atoms, *i.e*. 157.14 (6)°, with the other angles ranging from an acute 73.16 (6)°, for O1—Sn—O2, to 125.89 (9)°, being subtended by the two Sn-bound methyl groups.

The five-membered, SnON_2_C chelate ring is almost planar with a r.m.s. deviation of 0.0222 Å and in the same way, the six-membered, SnONC_3_ ring is close to planar with a r.m.s. deviation of 0.0155 Å; the dihedral angle between the chelate rings is small, being 2.90 (4)°. The bond lengths involving the nitro­gen atoms comprising the backbone of the chelate rings suggest some conjugation, *i.e*. N1—C1, N1—N2 and N2—C12 are 1.317 (3), 1.397 (2) and 1.303 (3) Å, respectively. The 10 atoms of the fused-ring system appended to the five-membered chelate ring make a dihedral angle of 2.01 (3)° with the chelate ring, a conformation allowing the formation of an intra­molecular hy­droxy-O—H⋯N(hydrazin­yl) hydrogen bond to close an *S*(6) loop, Table 2 ▸. The dihedral angle between the six-membered and fused benzene rings is 1.12 (5)°, indicating a strictly co-planar relationship. Significant planarity in the mol­ecule is indicated by the dihedral angle of 5.84 (4)° between the appended fused-ring system at C1 and the fused benzene ring. In addition, the dec­yloxy side chain has an all-*trans* conformation with the range of torsion angles being −174.96 (18)°, for C21—C22—C23—C24, to 179.79 (19)°, for C25—C26—C27—C28. Indeed, the r.m.s. deviation for the least-squares plane through all non-hydrogen atoms except the Sn-bound methyl groups is relatively small at 0.1179 Å, with maximum deviations being for the terminal methyl group of the alk­oxy chain, *i.e*. 0.296 (2) Å, and a central methyl­ene-C22 atom, *i.e*. 0.194 (2) Å. Hence, to a first approximation, the mol­ecule has mirror symmetry, relating the two Sn-bound methyl groups.

## Supra­molecular features   

**Figure 2:**
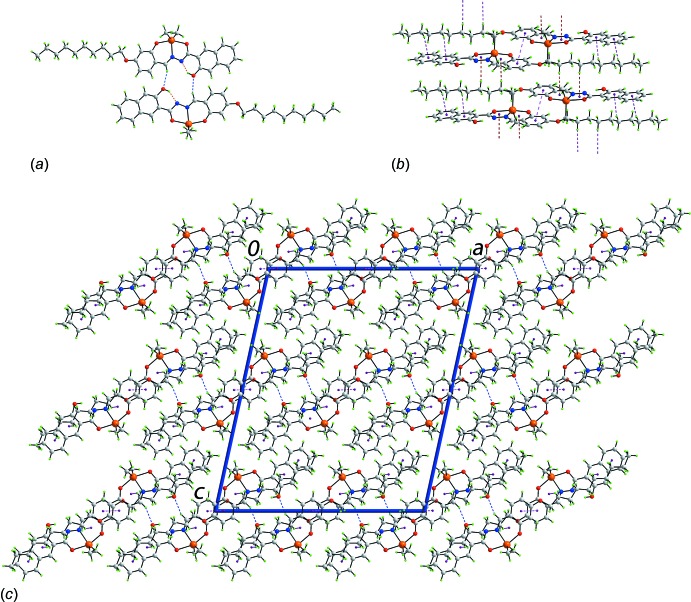
Mol­ecular packing in (I): (*a*) supra­molecular dimer sustained by imine-C—H⋯O(hy­droxy) inter­actions, shown as blue dashed lines, which incorporates two hy­droxy-O—H⋯N(hydrazin­yl) hydrogen bonds, shown as orange dashed lines, (*b*) view of a supra­molecular chain sustained by C—H⋯π inter­actions and (*c*) a view of the unit-cell contents in projection down the *b* axis, highlighting the stacking of supra­molecular layers along the *c* axis. The π—π, C—H⋯π(chelate ring) and C—H⋯π(arene) inter­actions are shown as pink, brown and purple dashed lines, respectively.

Aside from participating in an intra­molecular hy­droxy-O—H⋯N(hydrazin­yl) hydrogen bond, the hy­droxy-O atom accepts an inter­action from a centrosymmetrically-related imine-H atom, Table 2 ▸. This has the result that a 16-membered {⋯OC_3_N_2_CH}_2_ synthon is formed, which encapsulates two six-membered {⋯HOC_3_N} synthons formed by the intra­molecular hy­droxy-O—H⋯N(hydrazin­yl) hydrogen bonding mentioned above, Fig. 2 ▸ *a*. Centrosymmetrically related dimeric aggregates are linked *via* π–π inter­actions between dec­yloxy-substituted benzene rings [inter-centroid separation = 3.7724 (13) Å for symmetry operation: 1 − *x*, 1 − *y*, 1 − *z*]. The remaining inter­actions are of the type C—H⋯π and involve methyl­ene-C—H exclusively. While two of the inter­actions have benzene rings as acceptors, the other two have chelate rings as acceptors, *i.e*. are of the type C—H⋯π(chelate), a phenomenon gaining increasing attention (Tiekink, 2017 ▸); Table 2 ▸. Taken alone, the C—H⋯π inter­actions lead to supra­molecular chains as illustrated in Fig. 2 ▸ *b*. The result of all of the identified inter­molecular inter­actions is the formation of supra­molecular layers that stack along the *c* axis with no directional inter­actions between them, Fig. 2 ▸ *c*.

## Hirshfeld surface analysis   

**Figure 3:**
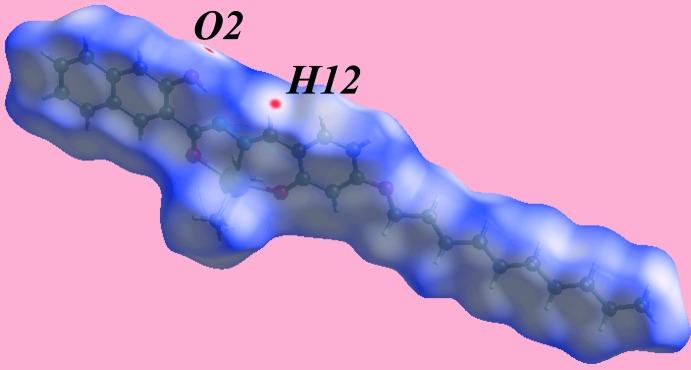
Hirshfeld surface for (I), mapped over *d*_norm_ in the range −0.053 to 1.621 au.

**Figure 4:**
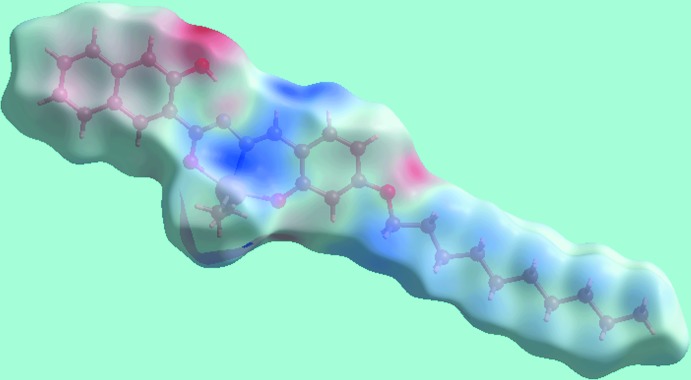
A view of Hirshfeld surface for (I), mapped over the electrostatic potential in the range ±0.075 au.

**Figure 5:**
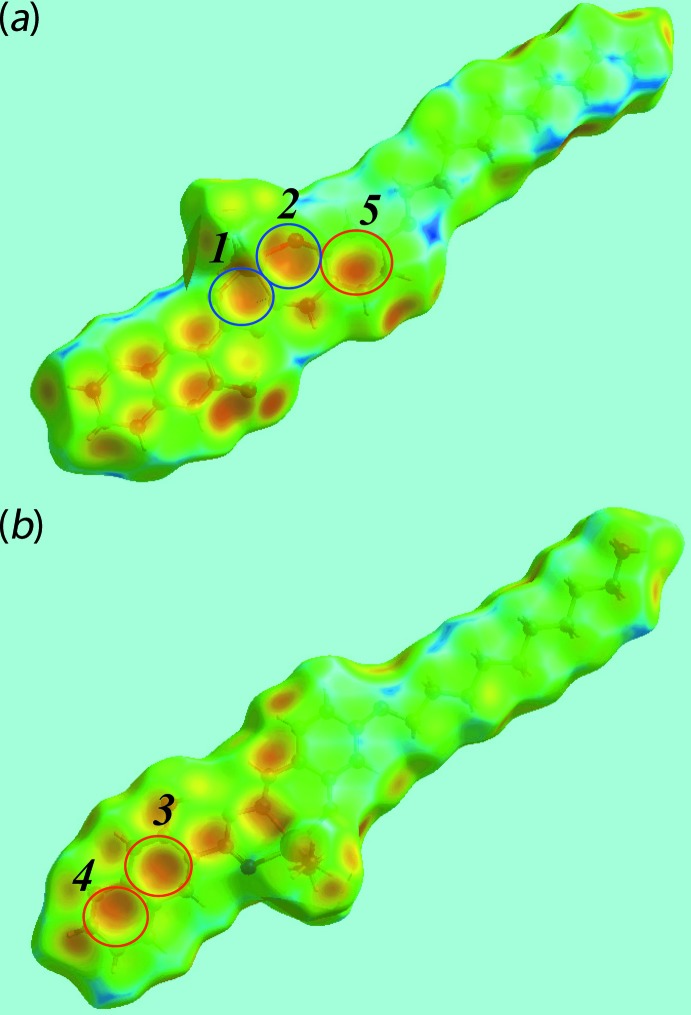
Two views of the Hirshfeld surface for (I) mapped over *d*_e_, showing inter­molecular C—H⋯π inter­actions involving the chelate and benzene rings of a reference mol­ecule highlighted with blue and red circles, respectively. Refer to Table 2 ▸ for designations of rings 1–4. Ring 5 comprises the (C13–C18) atoms.

**Figure 6:**
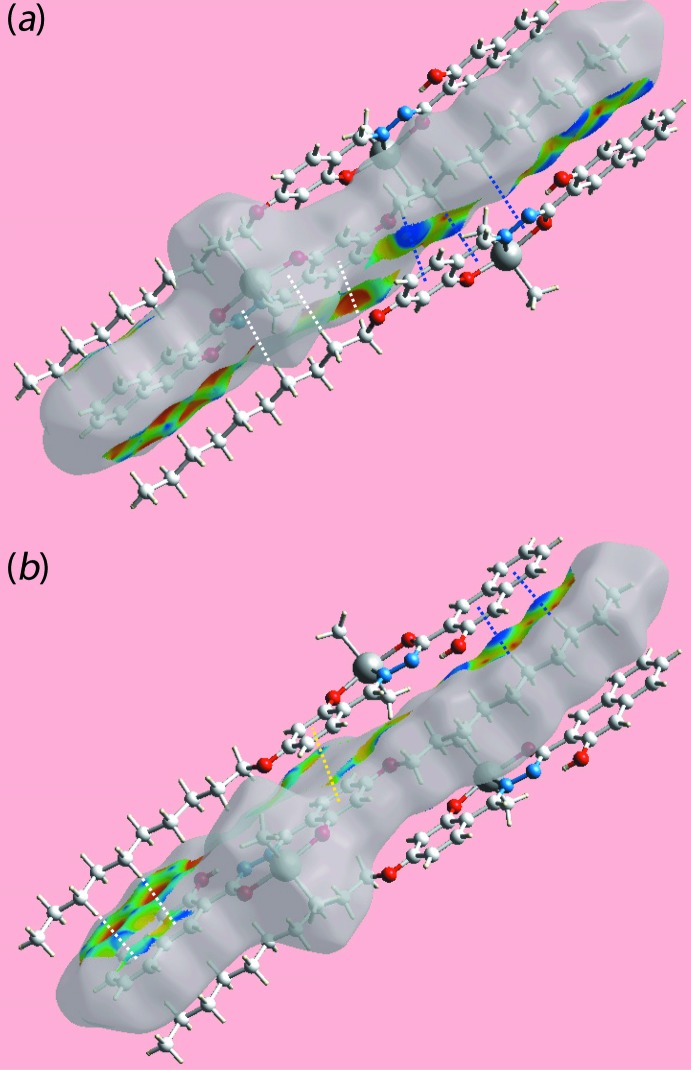
Two views of Hirshfeld surface for (I) mapped with shape-index property about a reference mol­ecule. The C—H⋯π and π⋯H—C inter­actions in both (*a*) and (*b*) are indicated with blue and white dotted lines, respectively. The yellow dotted lines in (*b*) indicate π–π stacking between benzene (C13–C18) rings.

The Hirshfeld surface analysis for (I) was performed as described in a recent publication of a related organotin structure (Mohamad *et al.*, 2017 ▸). From the view of the Hirshfeld surface mapped over *d*_norm_, in the range −0.053 to + 1.621 au, Fig. 3 ▸, the bright-red spots appearing near the hy­droxy-O2 and imine-H12 atoms represent the acceptor and donor of the inter­molecular C—H⋯O inter­action forming the {⋯OC_3_N_2_CH}_2_ synthon as discussed in the previous section; these are also viewed as blue and red regions near the H and O atoms on the Hirshfeld surface mapped over electrostatic potential (over the range ± 0.075 au), Fig. 4 ▸, corresponding to positive and negative potentials, respectively. In the absence of more conventional hydrogen bonds in the packing of (I), the structure contains two types of C—H⋯π inter­actions. The donors and acceptors of the C—H⋯π(arene) contacts are also viewed as respective light-blue and red regions on the Hirshfeld surface mapped over electrostatic potential, Fig. 4 ▸. In Fig. 5 ▸, the bright-orange spots enclosed within the circles around chelate (blue circle) and benzene (red) rings on the *d*_e_ mapped Hirshfeld surface, Fig. 5 ▸, illustrate all acceptors of the C—H⋯π contacts. The immediate environment about a reference mol­ecule within the Hirshfeld surface mapped with the shape-index property is illustrated in Fig. 6 ▸. The C—H⋯π(chelate) and C19—H19*A*⋯π(C13–C18) contacts at 1 − *x*, −*y*, 1 − *z* and their reciprocal contacts, *i.e*. π⋯H—C, are represented with blue and white dotted lines, respectively, in Fig. 6 ▸ *a*. The other C—H⋯π contacts involving benzene rings and π–π stacking inter­actions at 1 − *x*, 1 − *y*, 1 − *z* are illustrated in Fig. 6 ▸ *b*.

**Figure 7:**
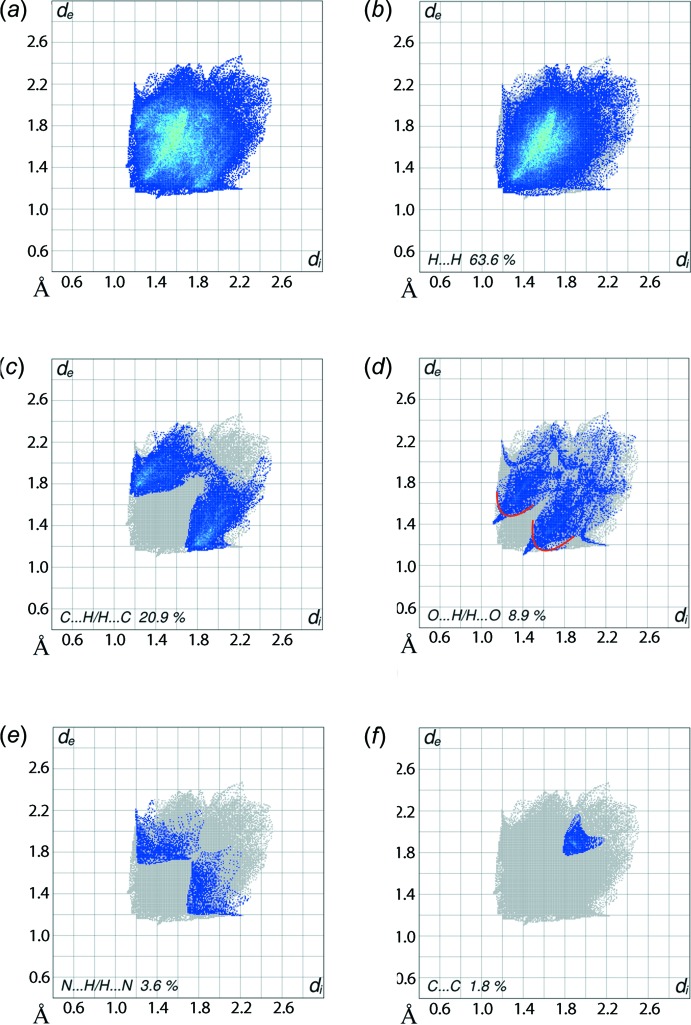
Fingerprint plots for (I): (*a*) overall and those delineated into (*b*) H⋯H, (*c*) C⋯H/H⋯C, (*d*) O⋯H/H⋯O, (*e*) N⋯H/H⋯N and (*f*) C⋯C contacts.

The overall two-dimensional fingerprint plot and those delineated into H⋯H, C⋯H/H⋯C, O⋯H/H⋯O, N⋯H/H⋯N and C⋯C contacts (McKinnon *et al.*, 2007 ▸) are illus­trated in Fig. 7 ▸ *a*–*f*; their relative contributions are summarized qu­anti­tatively in Table 3 ▸. The most notable observation from the Hirshfeld surface analysis of the structure of (I) is that hydrogen atoms are involved in the overwhelming majority of surface contacts, *i.e*. 97.0%.

A pair of very short peaks at *d*_e_ + *d*_i_ ∼ 2.38 Å in the fingerprint plot delineated into H⋯H contacts, Fig. 7 ▸ *b*, is due to a short inter­atomic contact between benzene-H18 and methyl­ene-H25*A* atoms, Table 4 ▸. The involvement of methyl­ene-H atoms in C—H⋯π inter­actions with the arene and chelate rings results in the second largest contribution to the overall Hirshfeld surface, *i.e*. 20.9%, in the form of C⋯H/H⋯C contacts, Fig. 7 ▸ *c*. The short inter­atomic C⋯H/H⋯C contact between the ring-C18 and methyl­ene-H19*A* atoms, Table 4 ▸, accounts for the presence of an inter­action between these atoms. Another short inter­atomic C⋯H/H⋯C contact, namely C10⋯H18 (Table 4 ▸), is merged in the corresponding plot of Fig. 7 ▸ *c*. The presence of two C—H⋯π(chelate) inter­actions, Table 2 ▸, can be easily recognized from the fingerprint plots delineated into C⋯H/H⋯C and N⋯H/H⋯N contacts, Fig. 7 ▸ *c* and *e*, as their ring centroids (*Cg*1 and *Cg*2; Table 2 ▸) are close to the N and C atoms of the chelate rings and so provide discernible contributions to the Hirshfeld surface. A recent study also confirmed the impact of C—H⋯π(chelate) inter­actions upon the Hirshfeld surface of a metal-organic compound (Jotani *et al.*, 2016 ▸). A pair of short spikes with tips at *d*_e_ + *d*_i_ ∼ 2.5 Å on the parabolic distribution of points around *d*_e_ + *d*_i_ ∼ 2.7 Å shown by a pair of red arcs in Fig. 7 ▸ *d* are the result of C—H⋯O and short inter­atomic O⋯H/H⋯O contacts, Table 4 ▸. A small but recognizable contribution, *i.e*. 1.8%, from C⋯C contacts to the Hirshfeld surface is assigned to π–π stacking inter­actions between symmetry-related (C13–C18) benzene rings, and appears as an arrow-like distribution of points around *d*_e_ = *d*_i_ ∼ 1.9 Å in Fig. 7 ▸ *f*. The other contacts, having low percentage contribution to the surface, are likely to have a negligible effect on the mol­ecular packing.

## Database survey   

**Figure 8:**
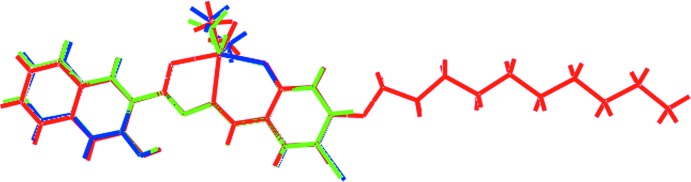
Overlap diagram of (I), red image, the 5-Cl analogue (green) and the 5-Br analogue (blue). The mol­ecules have been arranged so that the five-membered chelate rings are superimposed.

According to a search of the crystallographic literature (Groom *et al.*, 2016 ▸), there are approximately 100 diorganotin structures with Schiff base ligands having an O—C=N—N=C—C C—O backbone, as in (I). Of these, 13 have the 3-hy­droxy­naphthalene residue, reflecting the biological inter­est in these compounds (see *Chemical context*). Two di­methyl­tin structures are available with identical ligands apart from having a substituent in the 5-position, *i.e*. chloride (Lee *et al.*, 2009 ▸) and bromide (Lee *et al.*, 2010 ▸), rather than in the 4-position as for (I); the two halide structures are isostructural. An overlap diagram of (I) and the two 5-halide derivatives is shown in Fig. 8 ▸, which highlights the similarity between the structures. This borne out by the values of τ (Addison *et al.*, 1984 ▸), *i.e*. 0.47 and 0.46 for the chloride and bromide structures, respectively, *cf*. 0.52 for (I).

## Synthesis and crystallization   

All chemicals and solvents were used as purchased without purification, and all reactions were carried out under ambient conditions. The melting point was determined using an Electrothermal digital melting point apparatus and was uncorrected. The IR spectrum was obtained on a Perkin Elmer Spectrum 400 FT Mid-IR/Far-IR spectrophotometer from 4000 to 400 cm^−1^. The ^1^H NMR spectrum was recorded at room temperature in DMSO-*d*_6_ solution on a Jeol ECA 400 MHz FT–NMR spectrometer.

*N*-(4-Dec­oxy-2-oxido­benzyl­idene)-3-hy­droxy-2-napthohydrazide (1.0 mmol, 0.463 g) and tri­ethyl­amine (1.0 mmol, 0.14 ml) in ethyl acetate (25 ml) were added to di­methyl­tin dichloride (1.0 mmol, 0.220 g) in ethyl acetate (10 ml). The resulting mixture was stirred and refluxed for 3 h. The filtrate was evaporated until a precipitate was obtained. The precipitate was recrystallized from di­chloro­methane:di­methyl­formamide (1:1), and yellow prismatic crystals suitable for X-ray crystallographic studies were obtained from the slow evaporation of the filtrate. Yield: 0.366 g, 60%; M.p.: 507–508 K. IR (cm^−1^): 3162(*br*), 1633(*s*), 1597(*s*), 1169(*s*) cm^−1. 1^H NMR (in DMSO-*d*6): δ 11.34 (*s*, 1H, –OH), 8.57 (*s*, 1H, –N=CH), 6.25–6.40, 7.07–7.20 (*m*, 8H, aromatic-H), 8.47 (*s*, 1H, aromatic-H), 3.96 (*s*, 2H, –OCH_2_–), 1.28-1.82 (*m*, 16H, –CH_2_–), 0.91, (*s*, 6H, Sn—CH_3_), 0.89 (*s*, 3H, –CH_2_C*H*_3_).

## Refinement   

Crystal data, data collection and structure refinement details are summarized in Table 5 ▸. Carbon-bound H atoms were placed in calculated positions (C—H = 0.95–0.99 Å) and were included in the refinement in the riding-model approximation, with *U*_iso_(H) set to 1.2–1.5*U*_eq_(C). The oxygen-bound H atom was located in a difference Fourier map but was refined with a distance restraint of O—H = 0.84±0.01 Å, and with *U*_iso_(H) set to 1.5*U*_eq_(O). The maximum and minimum residual electron density peaks of 0.80 and 1.32 e Å^−3^ were located 0.42 and 0.83 Å, respectively, from the H23*B* and Sn atoms.

## Supplementary Material

Crystal structure: contains datablock(s) . DOI: 10.1107/S2056989017002365/hb7655sup1.cif

Structure factors: contains datablock(s) I. DOI: 10.1107/S2056989017002365/hb7655Isup2.hkl

CCDC reference: 1532445

Additional supporting information:  crystallographic information; 3D view; checkCIF report

## Figures and Tables

**Table 1:** Selected geometric parameters (Å, °)

Sn—O1	2.1600 (15)	Sn—C29	2.112 (2)
Sn—O3	2.0984 (15)	Sn—C30	2.106 (2)
Sn—N2	2.1503 (16)		
			
O1—Sn—O3	157.14 (6)	O3—Sn—C30	96.19 (8)
O1—Sn—N2	73.16 (6)	O3—Sn—C29	94.21 (8)
O1—Sn—C30	94.86 (8)	N2—Sn—C29	119.12 (8)
O1—Sn—C29	95.42 (8)	N2—Sn—C30	114.72 (8)
O3—Sn—N2	84.04 (6)	C29—Sn—C30	125.89 (9)

**Table 2:** Hydrogen-bond geometry (Å, °) *Cg*1–*Cg*4 are the centroids of the (Sn,O1,N1,N2,C1), (Sn,O3,N2,C12–C14), (C2–C4,C9–C11) and (C4—C9) rings, respectively.

*D*—H⋯*A*	*D*—H	H⋯*A*	*D*⋯*A*	*D*—H⋯*A*
O2—H2*O*⋯N1	0.83 (2)	1.86 (2)	2.580 (2)	145 (3)
C12—H12⋯O2^i^	0.95	2.52	3.386 (3)	152
C22—H22*A*⋯*Cg*1^ii^	0.99	2.86	3.782 (2)	155
C20—H20*B*⋯*Cg*2^ii^	0.99	2.76	3.650 (2)	149
C24—H24*B*⋯*Cg*3^iii^	0.99	2.74	3.609 (2)	146
C26—H26*B*⋯*Cg*4^iii^	0.99	2.78	3.696 (2)	154

Symmetry codes: (i) 
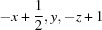
; (ii) 
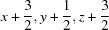
; (iii) 
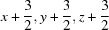
.

**Table 3:** Percentage contribution of the different inter­molecular contacts to the Hirshfeld surface in (I)

Contact	% contribution
H⋯H	63.6
C⋯H/H⋯C	20.9
O⋯H/H⋯O	8.9
N⋯H/H⋯N	3.6
C⋯C	1.8
C⋯O/O⋯C	1.1
O⋯O	0.1

**Table 4:** Short inter­atomic contacts in (I).

Contact	distance	symmetry operation
H18⋯H25*A*	2.38	− 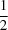 + *x*, −*y*, *z*
O2⋯H18	2.70	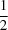 − *x*, *y*, 1 − *z*
C10⋯H18	2.83	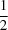 − *x*, *y*, 1 − *z*
C18⋯H19*A*	2.86	1 − *x*, −*y*, −1 + *z*

**Table 5:** Experimental details

Crystal data	
Chemical formula	[Sn(CH_3_)_2_(C_28_H_32_N_2_O_4_)]
*M*_r_	609.31
Crystal system, space group	Monoclinic, *I*2/*a*
Temperature (K)	100
*a*, *b*, *c* (Å)	25.2622 (9), 7.4543 (2), 29.9819 (11)
β (°)	102.349 (4)
*V* (Å^3^)	5515.3 (3)
*Z*	8
Radiation type	Mo *K*α
μ (mm^−1^)	0.96
Crystal size (mm)	0.26 × 0.21 × 0.09

Data collection	
Diffractometer	Rigaku SuperNova, Dual, Mo at zero, AtlasS2
Absorption correction	Multi-scan (*CrysAlis PRO*; Rigaku Oxford Diffraction, 2015 ▸)
*T*_min_, *T*_max_	0.756, 1.000
No. of measured, independent and observed [*I* > 2σ(*I*)] reflections	38191, 7182, 6371
*R*_int_	0.038
(sin θ/λ)_max_ (Å^−1^)	0.696

Refinement	
*R*[*F* ^2^ > 2σ(*F* ^2^)], *wR*(*F* ^2^), *S*	0.031, 0.076, 1.01
No. of reflections	7182
No. of parameters	340
No. of restraints	1
Δρ_max_, Δρ_min_ (e Å^−3^)	0.80, −1.32

Computer programs: *CrysAlis PRO* (Rigaku Oxford Diffraction, 2015 ▸), *SHELXS* (Sheldrick, 2008 ▸), *SHELXL2014* (Sheldrick, 2015 ▸), *ORTEP-3 for Windows* (Farrugia, 2012 ▸), *QMol* (Gans & Shalloway, 2001 ▸) and *DIAMOND* (Brandenburg, 2006 ▸), *publCIF* (Westrip, 2010 ▸).

## supplementary crystallographic information

### Crystal data

**Table:**

[Sn(CH_3_)_2_(C_28_H_32_N_2_O_4_)]	*F*(000) = 2512
*M**_r_* = 609.31	*D*_x_ = 1.468 Mg m^−^^3^
Monoclinic, *I*2/*a*	Mo *K*α radiation, λ = 0.71073 Å
*a* = 25.2622 (9) Å	Cell parameters from 14600 reflections
*b* = 7.4543 (2) Å	θ = 2.9–29.3°
*c* = 29.9819 (11) Å	µ = 0.96 mm^−^^1^
β = 102.349 (4)°	*T* = 100 K
*V* = 5515.3 (3) Å^3^	Prism, yellow
*Z* = 8	0.26 × 0.21 × 0.09 mm

### Data collection

**Table:**

Rigaku SuperNova, Dual, Mo at zero, AtlasS2 diffractometer	7182 independent reflections
Radiation source: micro-focus sealed X-ray tube, SuperNova (Mo) X-ray Source	6371 reflections with *I* > 2σ(*I*)
Mirror monochromator	*R*_int_ = 0.038
ω scans	θ_max_ = 29.7°, θ_min_ = 2.8°
Absorption correction: multi-scan (CrysAlis PRO; Rigaku Oxford Diffraction, 2015)	*h* = −33→34
*T*_min_ = 0.756, *T*_max_ = 1.000	*k* = −10→9
38191 measured reflections	*l* = −40→41

### Refinement

**Table:**

Refinement on *F*^2^	1 restraint
Least-squares matrix: full	Hydrogen site location: mixed
*R*[*F*^2^ > 2σ(*F*^2^)] = 0.031	*w* = 1/[σ^2^(*F*_o_^2^) + (0.0376*P*)^2^ + 14.285*P*] where *P* = (*F*_o_^2^ + 2*F*_c_^2^)/3
*wR*(*F*^2^) = 0.076	(Δ/σ)_max_ = 0.006
*S* = 1.01	Δρ_max_ = 0.80 e Å^−^^3^
7182 reflections	Δρ_min_ = −1.32 e Å^−^^3^
340 parameters	

### Special details

**Table:**

Geometry. All esds (except the esd in the dihedral angle between two l.s. planes) are estimated using the full covariance matrix. The cell esds are taken into account individually in the estimation of esds in distances, angles and torsion angles; correlations between esds in cell parameters are only used when they are defined by crystal symmetry. An approximate (isotropic) treatment of cell esds is used for estimating esds involving l.s. planes.

### Fractional atomic coordinates and isotropic or equivalent isotropic displacement parameters (Å^2^)

**Table:**

	*x*	*y*	*z*	*U*_iso_*/*U*_eq_	
Sn	0.42928 (2)	0.37591 (2)	0.63968 (2)	0.01137 (5)	
O1	0.35465 (6)	0.4300 (2)	0.66140 (5)	0.0167 (3)	
O2	0.21397 (6)	0.5277 (2)	0.55907 (5)	0.0183 (3)	
H2O	0.2451 (6)	0.506 (4)	0.5555 (10)	0.027*	
O3	0.47942 (6)	0.3222 (2)	0.59379 (5)	0.0181 (3)	
O4	0.54342 (6)	0.2034 (2)	0.45789 (5)	0.0201 (3)	
N1	0.31505 (7)	0.4446 (2)	0.58481 (6)	0.0129 (3)	
N2	0.36668 (7)	0.3985 (2)	0.57883 (6)	0.0110 (3)	
C1	0.31320 (8)	0.4565 (3)	0.62829 (7)	0.0124 (4)	
C2	0.26038 (8)	0.5020 (3)	0.63921 (7)	0.0129 (4)	
C3	0.25664 (8)	0.5125 (3)	0.68442 (7)	0.0133 (4)	
H3	0.2883	0.4944	0.7076	0.016*	
C4	0.20706 (8)	0.5496 (3)	0.69711 (7)	0.0146 (4)	
C5	0.20297 (9)	0.5598 (3)	0.74350 (7)	0.0188 (4)	
H5	0.2345	0.5437	0.7669	0.023*	
C6	0.15430 (9)	0.5926 (3)	0.75491 (8)	0.0214 (5)	
H6	0.1522	0.6003	0.7861	0.026*	
C7	0.10712 (9)	0.6150 (3)	0.72021 (8)	0.0204 (5)	
H7	0.0733	0.6357	0.7284	0.025*	
C8	0.10947 (9)	0.6074 (3)	0.67508 (8)	0.0178 (4)	
H8	0.0774	0.6232	0.6522	0.021*	
C9	0.15978 (8)	0.5758 (3)	0.66226 (7)	0.0146 (4)	
C10	0.16431 (8)	0.5697 (3)	0.61610 (7)	0.0150 (4)	
H10	0.1331	0.5913	0.5927	0.018*	
C11	0.21280 (8)	0.5332 (3)	0.60440 (7)	0.0134 (4)	
C12	0.37054 (8)	0.3749 (3)	0.53660 (7)	0.0121 (4)	
H12	0.3383	0.3905	0.5140	0.014*	
C13	0.41771 (8)	0.3285 (3)	0.52079 (7)	0.0126 (4)	
C14	0.47001 (8)	0.3039 (3)	0.54913 (7)	0.0131 (4)	
C15	0.51337 (8)	0.2588 (3)	0.52845 (7)	0.0143 (4)	
H15	0.5486	0.2403	0.5468	0.017*	
C16	0.50482 (8)	0.2413 (3)	0.48147 (7)	0.0144 (4)	
C17	0.45295 (9)	0.2635 (3)	0.45328 (7)	0.0173 (4)	
H17	0.4476	0.2495	0.4211	0.021*	
C18	0.41073 (8)	0.3053 (3)	0.47295 (7)	0.0154 (4)	
H18	0.3756	0.3194	0.4541	0.018*	
C19	0.59810 (8)	0.1635 (3)	0.48078 (7)	0.0152 (4)	
H19A	0.5993	0.0624	0.5024	0.018*	
H19B	0.6158	0.2694	0.4976	0.018*	
C20	0.62486 (9)	0.1133 (3)	0.44178 (7)	0.0152 (4)	
H20A	0.6187	0.2127	0.4193	0.018*	
H20B	0.6060	0.0064	0.4264	0.018*	
C21	0.68525 (8)	0.0733 (3)	0.45358 (7)	0.0160 (4)	
H21A	0.6923	−0.0341	0.4734	0.019*	
H21B	0.7051	0.1759	0.4703	0.019*	
C22	0.70459 (8)	0.0402 (3)	0.40919 (7)	0.0159 (4)	
H22A	0.6830	−0.0595	0.3927	0.019*	
H22B	0.6964	0.1487	0.3899	0.019*	
C23	0.76433 (8)	−0.0045 (3)	0.41369 (7)	0.0156 (4)	
H23A	0.7726	−0.1202	0.4299	0.019*	
H23B	0.7868	0.0896	0.4319	0.019*	
C24	0.77803 (8)	−0.0168 (3)	0.36659 (7)	0.0152 (4)	
H24A	0.7549	−0.1105	0.3488	0.018*	
H24B	0.7687	0.0988	0.3506	0.018*	
C25	0.83719 (9)	−0.0601 (3)	0.36696 (7)	0.0167 (4)	
H25A	0.8465	−0.1774	0.3821	0.020*	
H25B	0.8606	0.0321	0.3851	0.020*	
C26	0.84886 (8)	−0.0668 (3)	0.31918 (7)	0.0151 (4)	
H26A	0.8271	−0.1642	0.3017	0.018*	
H26B	0.8372	0.0477	0.3034	0.018*	
C27	0.90843 (9)	−0.0980 (3)	0.31902 (7)	0.0174 (4)	
H27A	0.9303	−0.0004	0.3362	0.021*	
H27B	0.9203	−0.2125	0.3348	0.021*	
C28	0.91884 (9)	−0.1048 (3)	0.27092 (8)	0.0207 (5)	
H28A	0.8977	−0.2027	0.2539	0.031*	
H28B	0.9575	−0.1255	0.2725	0.031*	
H28C	0.9080	0.0093	0.2554	0.031*	
C29	0.44335 (10)	0.1248 (3)	0.67269 (8)	0.0199 (4)	
H29A	0.4669	0.0522	0.6578	0.030*	
H29B	0.4088	0.0626	0.6708	0.030*	
H29C	0.4609	0.1430	0.7048	0.030*	
C30	0.46626 (9)	0.6203 (3)	0.66410 (8)	0.0199 (4)	
H30A	0.5018	0.5964	0.6838	0.030*	
H30B	0.4434	0.6835	0.6816	0.030*	
H30C	0.4708	0.6948	0.6382	0.030*	

### Atomic displacement parameters (Å^2^)

**Table:**

	*U*^11^	*U*^22^	*U*^33^	*U*^12^	*U*^13^	*U*^23^
Sn	0.00732 (7)	0.01518 (8)	0.01046 (7)	0.00087 (5)	−0.00065 (5)	−0.00112 (5)
O1	0.0075 (6)	0.0288 (8)	0.0121 (7)	0.0020 (6)	−0.0015 (5)	−0.0006 (6)
O2	0.0108 (7)	0.0300 (9)	0.0132 (7)	0.0026 (6)	0.0004 (6)	−0.0029 (6)
O3	0.0100 (7)	0.0330 (9)	0.0103 (7)	0.0025 (6)	0.0002 (5)	−0.0025 (6)
O4	0.0116 (7)	0.0340 (9)	0.0150 (7)	0.0052 (7)	0.0033 (6)	−0.0020 (7)
N1	0.0056 (7)	0.0174 (8)	0.0150 (8)	0.0014 (7)	0.0007 (6)	−0.0009 (7)
N2	0.0063 (7)	0.0129 (8)	0.0127 (8)	0.0002 (6)	−0.0006 (6)	−0.0008 (6)
C1	0.0101 (9)	0.0116 (9)	0.0139 (9)	−0.0010 (7)	−0.0008 (7)	−0.0007 (7)
C2	0.0099 (9)	0.0135 (9)	0.0143 (9)	−0.0008 (7)	0.0004 (7)	−0.0007 (8)
C3	0.0097 (9)	0.0151 (10)	0.0143 (9)	−0.0006 (7)	0.0006 (7)	−0.0010 (8)
C4	0.0110 (9)	0.0159 (10)	0.0165 (9)	−0.0034 (8)	0.0023 (7)	−0.0013 (8)
C5	0.0148 (10)	0.0235 (11)	0.0176 (10)	−0.0014 (9)	0.0023 (8)	−0.0008 (9)
C6	0.0190 (11)	0.0272 (12)	0.0199 (11)	−0.0043 (9)	0.0086 (9)	−0.0036 (9)
C7	0.0132 (10)	0.0219 (11)	0.0285 (12)	−0.0017 (9)	0.0097 (9)	−0.0026 (9)
C8	0.0093 (9)	0.0189 (11)	0.0244 (11)	0.0006 (8)	0.0020 (8)	−0.0018 (9)
C9	0.0099 (9)	0.0133 (9)	0.0200 (10)	−0.0020 (8)	0.0015 (8)	−0.0013 (8)
C10	0.0094 (9)	0.0166 (10)	0.0170 (10)	−0.0003 (8)	−0.0019 (7)	−0.0021 (8)
C11	0.0132 (9)	0.0117 (10)	0.0142 (9)	−0.0006 (7)	0.0004 (7)	−0.0021 (7)
C12	0.0101 (9)	0.0124 (9)	0.0126 (9)	−0.0014 (7)	−0.0001 (7)	−0.0002 (7)
C13	0.0117 (9)	0.0125 (9)	0.0128 (9)	−0.0001 (7)	0.0008 (7)	0.0004 (7)
C14	0.0114 (9)	0.0142 (9)	0.0129 (9)	−0.0006 (8)	0.0005 (7)	−0.0012 (8)
C15	0.0098 (9)	0.0173 (10)	0.0152 (9)	0.0006 (8)	0.0013 (7)	−0.0004 (8)
C16	0.0126 (9)	0.0146 (10)	0.0168 (10)	0.0011 (8)	0.0050 (8)	−0.0008 (8)
C17	0.0143 (10)	0.0237 (11)	0.0131 (9)	0.0004 (8)	0.0010 (8)	−0.0010 (8)
C18	0.0121 (9)	0.0198 (10)	0.0128 (9)	0.0012 (8)	−0.0004 (7)	0.0012 (8)
C19	0.0107 (9)	0.0188 (10)	0.0158 (9)	0.0023 (8)	0.0027 (7)	−0.0007 (8)
C20	0.0138 (10)	0.0176 (10)	0.0145 (9)	0.0010 (8)	0.0038 (8)	0.0017 (8)
C21	0.0115 (9)	0.0198 (10)	0.0171 (10)	0.0011 (8)	0.0037 (8)	0.0000 (8)
C22	0.0134 (10)	0.0167 (10)	0.0179 (10)	0.0005 (8)	0.0043 (8)	0.0006 (8)
C23	0.0117 (9)	0.0187 (10)	0.0163 (9)	0.0008 (8)	0.0031 (8)	0.0019 (8)
C24	0.0122 (9)	0.0168 (10)	0.0163 (9)	0.0003 (8)	0.0025 (8)	−0.0009 (8)
C25	0.0136 (10)	0.0197 (10)	0.0171 (10)	0.0032 (8)	0.0037 (8)	0.0021 (9)
C26	0.0127 (9)	0.0175 (10)	0.0151 (9)	0.0025 (8)	0.0034 (8)	−0.0012 (8)
C27	0.0141 (10)	0.0213 (11)	0.0172 (10)	0.0039 (8)	0.0045 (8)	−0.0008 (8)
C28	0.0165 (11)	0.0272 (12)	0.0193 (10)	0.0053 (9)	0.0056 (8)	−0.0006 (9)
C29	0.0209 (11)	0.0200 (11)	0.0186 (10)	0.0062 (9)	0.0036 (8)	0.0030 (9)
C30	0.0171 (10)	0.0190 (11)	0.0222 (11)	−0.0015 (9)	0.0010 (8)	−0.0036 (9)

### Geometric parameters (Å, º)

**Table:**

Sn—O1	2.1600 (15)	C17—C18	1.361 (3)
Sn—O3	2.0984 (15)	C17—H17	0.9500
Sn—N2	2.1503 (16)	C18—H18	0.9500
Sn—C29	2.112 (2)	C19—C20	1.517 (3)
Sn—C30	2.106 (2)	C19—H19A	0.9900
O1—C1	1.295 (2)	C19—H19B	0.9900
O2—C11	1.366 (2)	C20—C21	1.520 (3)
O2—H2O	0.833 (10)	C20—H20A	0.9900
O3—C14	1.316 (2)	C20—H20B	0.9900
O4—C16	1.351 (2)	C21—C22	1.532 (3)
O4—C19	1.436 (2)	C21—H21A	0.9900
N1—C1	1.317 (3)	C21—H21B	0.9900
N1—N2	1.397 (2)	C22—C23	1.523 (3)
N2—C12	1.303 (3)	C22—H22A	0.9900
C1—C2	1.480 (3)	C22—H22B	0.9900
C2—C3	1.381 (3)	C23—C24	1.527 (3)
C2—C11	1.432 (3)	C23—H23A	0.9900
C3—C4	1.412 (3)	C23—H23B	0.9900
C3—H3	0.9500	C24—C25	1.527 (3)
C4—C9	1.422 (3)	C24—H24A	0.9900
C4—C5	1.419 (3)	C24—H24B	0.9900
C5—C6	1.367 (3)	C25—C26	1.524 (3)
C5—H5	0.9500	C25—H25A	0.9900
C6—C7	1.414 (3)	C25—H25B	0.9900
C6—H6	0.9500	C26—C27	1.524 (3)
C7—C8	1.368 (3)	C26—H26A	0.9900
C7—H7	0.9500	C26—H26B	0.9900
C8—C9	1.424 (3)	C27—C28	1.521 (3)
C8—H8	0.9500	C27—H27A	0.9900
C9—C10	1.414 (3)	C27—H27B	0.9900
C10—C11	1.372 (3)	C28—H28A	0.9800
C10—H10	0.9500	C28—H28B	0.9800
C12—C13	1.416 (3)	C28—H28C	0.9800
C12—H12	0.9500	C29—H29A	0.9800
C13—C14	1.421 (3)	C29—H29B	0.9800
C13—C18	1.418 (3)	C29—H29C	0.9800
C14—C15	1.410 (3)	C30—H30A	0.9800
C15—C16	1.385 (3)	C30—H30B	0.9800
C15—H15	0.9500	C30—H30C	0.9800
C16—C17	1.409 (3)		
			
O1—Sn—O3	157.14 (6)	O4—C19—C20	102.98 (16)
O1—Sn—N2	73.16 (6)	O4—C19—H19A	111.2
O1—Sn—C30	94.86 (8)	C20—C19—H19A	111.2
O1—Sn—C29	95.42 (8)	O4—C19—H19B	111.2
O3—Sn—N2	84.04 (6)	C20—C19—H19B	111.2
O3—Sn—C30	96.19 (8)	H19A—C19—H19B	109.1
O3—Sn—C29	94.21 (8)	C19—C20—C21	117.36 (18)
N2—Sn—C29	119.12 (8)	C19—C20—H20A	108.0
N2—Sn—C30	114.72 (8)	C21—C20—H20A	108.0
C29—Sn—C30	125.89 (9)	C19—C20—H20B	108.0
C1—O1—Sn	114.34 (13)	C21—C20—H20B	108.0
C11—O2—H2O	111 (2)	H20A—C20—H20B	107.2
C14—O3—Sn	133.15 (13)	C20—C21—C22	108.66 (17)
C16—O4—C19	121.44 (16)	C20—C21—H21A	110.0
C1—N1—N2	112.04 (16)	C22—C21—H21A	110.0
C12—N2—N1	115.10 (16)	C20—C21—H21B	110.0
C12—N2—Sn	128.31 (14)	C22—C21—H21B	110.0
N1—N2—Sn	116.60 (12)	H21A—C21—H21B	108.3
O1—C1—N1	123.67 (18)	C23—C22—C21	116.88 (17)
O1—C1—C2	119.01 (17)	C23—C22—H22A	108.1
N1—C1—C2	117.32 (17)	C21—C22—H22A	108.1
C3—C2—C11	118.88 (18)	C23—C22—H22B	108.1
C3—C2—C1	118.98 (18)	C21—C22—H22B	108.1
C11—C2—C1	122.14 (18)	H22A—C22—H22B	107.3
C2—C3—C4	121.77 (19)	C22—C23—C24	110.33 (17)
C2—C3—H3	119.1	C22—C23—H23A	109.6
C4—C3—H3	119.1	C24—C23—H23A	109.6
C3—C4—C9	118.88 (19)	C22—C23—H23B	109.6
C3—C4—C5	121.97 (19)	C24—C23—H23B	109.6
C9—C4—C5	119.14 (19)	H23A—C23—H23B	108.1
C6—C5—C4	120.9 (2)	C25—C24—C23	114.90 (17)
C6—C5—H5	119.6	C25—C24—H24A	108.5
C4—C5—H5	119.6	C23—C24—H24A	108.5
C5—C6—C7	119.9 (2)	C25—C24—H24B	108.5
C5—C6—H6	120.0	C23—C24—H24B	108.5
C7—C6—H6	120.0	H24A—C24—H24B	107.5
C8—C7—C6	121.0 (2)	C24—C25—C26	112.70 (17)
C8—C7—H7	119.5	C24—C25—H25A	109.1
C6—C7—H7	119.5	C26—C25—H25A	109.1
C7—C8—C9	120.3 (2)	C24—C25—H25B	109.1
C7—C8—H8	119.9	C26—C25—H25B	109.1
C9—C8—H8	119.9	H25A—C25—H25B	107.8
C4—C9—C10	118.93 (19)	C27—C26—C25	113.44 (17)
C4—C9—C8	118.8 (2)	C27—C26—H26A	108.9
C10—C9—C8	122.23 (19)	C25—C26—H26A	108.9
C11—C10—C9	121.36 (19)	C27—C26—H26B	108.9
C11—C10—H10	119.3	C25—C26—H26B	108.9
C9—C10—H10	119.3	H26A—C26—H26B	107.7
O2—C11—C10	118.09 (18)	C28—C27—C26	112.29 (18)
O2—C11—C2	121.78 (18)	C28—C27—H27A	109.1
C10—C11—C2	120.13 (18)	C26—C27—H27A	109.1
N2—C12—C13	126.98 (18)	C28—C27—H27B	109.1
N2—C12—H12	116.5	C26—C27—H27B	109.1
C13—C12—H12	116.5	H27A—C27—H27B	107.9
C14—C13—C12	124.92 (18)	C27—C28—H28A	109.5
C14—C13—C18	119.19 (18)	C27—C28—H28B	109.5
C12—C13—C18	115.89 (18)	H28A—C28—H28B	109.5
O3—C14—C15	118.94 (18)	C27—C28—H28C	109.5
O3—C14—C13	122.50 (18)	H28A—C28—H28C	109.5
C15—C14—C13	118.57 (18)	H28B—C28—H28C	109.5
C16—C15—C14	120.18 (19)	Sn—C29—H29A	109.5
C16—C15—H15	119.9	Sn—C29—H29B	109.5
C14—C15—H15	119.9	H29A—C29—H29B	109.5
O4—C16—C15	125.36 (19)	Sn—C29—H29C	109.5
O4—C16—C17	113.16 (18)	H29A—C29—H29C	109.5
C15—C16—C17	121.48 (19)	H29B—C29—H29C	109.5
C18—C17—C16	118.78 (19)	Sn—C30—H30A	109.5
C18—C17—H17	120.6	Sn—C30—H30B	109.5
C16—C17—H17	120.6	H30A—C30—H30B	109.5
C17—C18—C13	121.79 (19)	Sn—C30—H30C	109.5
C17—C18—H18	119.1	H30A—C30—H30C	109.5
C13—C18—H18	119.1	H30B—C30—H30C	109.5
			
C1—N1—N2—C12	176.40 (18)	C1—C2—C11—C10	178.0 (2)
C1—N1—N2—Sn	−3.7 (2)	N1—N2—C12—C13	179.75 (19)
Sn—O1—C1—N1	2.8 (3)	Sn—N2—C12—C13	−0.2 (3)
Sn—O1—C1—C2	−177.56 (14)	N2—C12—C13—C14	−1.6 (3)
N2—N1—C1—O1	0.6 (3)	N2—C12—C13—C18	178.1 (2)
N2—N1—C1—C2	−179.12 (17)	Sn—O3—C14—C15	−177.01 (15)
O1—C1—C2—C3	−0.8 (3)	Sn—O3—C14—C13	3.2 (3)
N1—C1—C2—C3	178.86 (19)	C12—C13—C14—O3	0.2 (3)
O1—C1—C2—C11	179.99 (19)	C18—C13—C14—O3	−179.6 (2)
N1—C1—C2—C11	−0.3 (3)	C12—C13—C14—C15	−179.6 (2)
C11—C2—C3—C4	1.3 (3)	C18—C13—C14—C15	0.6 (3)
C1—C2—C3—C4	−177.91 (19)	O3—C14—C15—C16	−179.0 (2)
C2—C3—C4—C9	0.3 (3)	C13—C14—C15—C16	0.8 (3)
C2—C3—C4—C5	179.7 (2)	C19—O4—C16—C15	4.8 (3)
C3—C4—C5—C6	−178.8 (2)	C19—O4—C16—C17	−175.49 (19)
C9—C4—C5—C6	0.7 (3)	C14—C15—C16—O4	178.2 (2)
C4—C5—C6—C7	0.6 (4)	C14—C15—C16—C17	−1.5 (3)
C5—C6—C7—C8	−1.1 (4)	O4—C16—C17—C18	−178.9 (2)
C6—C7—C8—C9	0.2 (3)	C15—C16—C17—C18	0.9 (3)
C3—C4—C9—C10	−2.0 (3)	C16—C17—C18—C13	0.5 (3)
C5—C4—C9—C10	178.5 (2)	C14—C13—C18—C17	−1.3 (3)
C3—C4—C9—C8	178.0 (2)	C12—C13—C18—C17	178.9 (2)
C5—C4—C9—C8	−1.5 (3)	C16—O4—C19—C20	174.88 (18)
C7—C8—C9—C4	1.0 (3)	O4—C19—C20—C21	176.30 (18)
C7—C8—C9—C10	−179.0 (2)	C19—C20—C21—C22	−175.71 (18)
C4—C9—C10—C11	2.2 (3)	C20—C21—C22—C23	−179.36 (18)
C8—C9—C10—C11	−177.8 (2)	C21—C22—C23—C24	−174.96 (18)
C9—C10—C11—O2	179.35 (19)	C22—C23—C24—C25	179.63 (18)
C9—C10—C11—C2	−0.6 (3)	C23—C24—C25—C26	−178.75 (18)
C3—C2—C11—O2	178.89 (19)	C24—C25—C26—C27	176.35 (18)
C1—C2—C11—O2	−1.9 (3)	C25—C26—C27—C28	179.79 (19)
C3—C2—C11—C10	−1.2 (3)		

### Hydrogen-bond geometry (Å, º)

*Cg*1–*Cg*4 are the centroids of the (Sn,O1,N1,N2,C1), (Sn,O3,N2,C12–C14), (C2–C4,C9–C11) and (C4—C9) rings, respectively.

**Table:**

*D*—H···*A*	*D*—H	H···*A*	*D*···*A*	*D*—H···*A*
O2—H2*O*···N1	0.83 (2)	1.86 (2)	2.580 (2)	145 (3)
C12—H12···O2^i^	0.95	2.52	3.386 (3)	152
C22—H22*A*···*Cg*1^ii^	0.99	2.86	3.782 (2)	155
C20—H20*B*···*Cg*2^ii^	0.99	2.76	3.650 (2)	149
C24—H24*B*···*Cg*3^iii^	0.99	2.74	3.609 (2)	146
C26—H26*B*···*Cg*4^iii^	0.99	2.78	3.696 (2)	154

Symmetry codes: (i) −*x*+1/2, *y*, −*z*+1; (ii) *x*+3/2, *y*+1/2, *z*+3/2; (iii) *x*+3/2, *y*+3/2, *z*+3/2.
